# An efficient algorithmic approach for mass spectrometry-based disulfide connectivity determination using multi-ion analysis

**DOI:** 10.1186/1471-2105-12-S1-S12

**Published:** 2011-02-15

**Authors:** William Murad, Rahul Singh, Ten-Yang Yen

**Affiliations:** 1 Department of Computer Science, San Francisco State University, 1600 Holloway Avenue, San Francisco, CA, 94132, USA; 2 Department of Chemistry and Biochemistry, San Francisco State University, 1600 Holloway Avenue, San Francisco, CA, 94132, USA

## Abstract

**Background:**

Determining the disulfide (S-S) bond pattern in a protein is often crucial for understanding its structure and function. In recent research, mass spectrometry (MS) based analysis has been applied to this problem following protein digestion under both partial reduction and non-reduction conditions. However, this paradigm still awaits solutions to certain algorithmic problems fundamental amongst which is the efficient matching of an exponentially growing set of putative S-S bonded structural alternatives to the large amounts of experimental spectrometric data. Current methods circumvent this challenge primarily through simplifications, such as by assuming only the occurrence of certain ion-types (*b*-ions and *y*-ions) that predominate in the more popular dissociation methods, such as collision-induced dissociation (*CID*). Unfortunately, this can adversely impact the quality of results.

**Method:**

We present an algorithmic approach to this problem that can, with high computational efficiency, analyze multiple ions types (*a*, *b*, b^o^, b^*^, c, *x*, y, y^o^, y^*^, and *z*) and deal with complex bonding topologies, such as inter/intra bonding involving more than two peptides. The proposed approach combines an approximation algorithm-based search formulation with data driven parameter estimation. This formulation considers only those regions of the search space where the correct solution resides with a high likelihood. Putative disulfide bonds thus obtained are finally combined in a globally consistent pattern to yield the overall disulfide bonding topology of the molecule. Additionally, each bond is associated with a confidence score, which aids in interpretation and assimilation of the results.

**Results:**

The method was tested on nine different eukaryotic Glycosyltransferases possessing disulfide bonding topologies of varying complexity. Its performance was found to be characterized by high efficiency (in terms of time and the fraction of search space considered), sensitivity, specificity, and accuracy. The method was also compared with other techniques at the state-of-the-art. It was found to perform as well or better than the competing techniques. An implementation is available at: http://tintin.sfsu.edu/~whemurad/disulfidebond.

**Conclusions:**

This research addresses some of the significant challenges in MS-based disulfide bond determination. To the best of our knowledge, this is the first algorithmic work that can consider multiple ion types in this problem setting while simultaneously ensuring polynomial time complexity and high accuracy of results.

## Background

**Figure 1:**
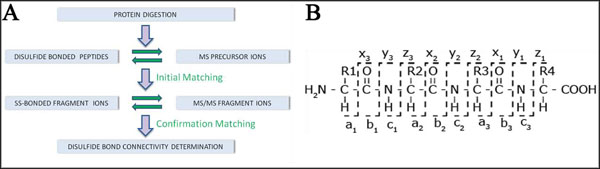
**MS-based approach diagrammatic representation.** (A) Once a protein is digested, the theoretically possible disulfide bonded peptides are compared with experimentally obtained precursor ions. In order to confirm each correspondence, the possible disulfide bonded fragment ions are next compared with experimentally generated MS/MS spectra. (B) Most of the different fragment ions (and their nomenclature) that can be observed. Ions types not represented here include b and y ions which have either lost a water molecule (b^o^, y^o^) or have lost an ammonia molecule (b^*^, y^*^).

Disulfide (S-S) bonds are known to play an important role in protein structure and function. Among others, this includes: influencing protein folding and stabilization, formation of characteristic structural motifs such as the cysteine knot, mediation of thiol-disulfide interchange reactions, and regulation of enzymatic activity. Early computational approaches for S-S bond determination focused on two learning-driven formulations based on the protein primary structure [1]: *residue classification* (distinguish bonded and free cysteines) and *connectivity prediction* (determine the S-S connectivity pattern). In recent times, the increasing availability and accuracy of mass spectrometry [2] (MS) has opened up an alternate approach; its essence lies in matching the theoretical spectra of ionized peptide fragments with experimentally obtained spectra to identify the presence of specific S-S bonds. A diagrammatic representation of the key steps of a MS-based approach is presented in Figure 1, along with the different types of fragment ions that can be generated as an outcome of this process.

**Figure 2:**
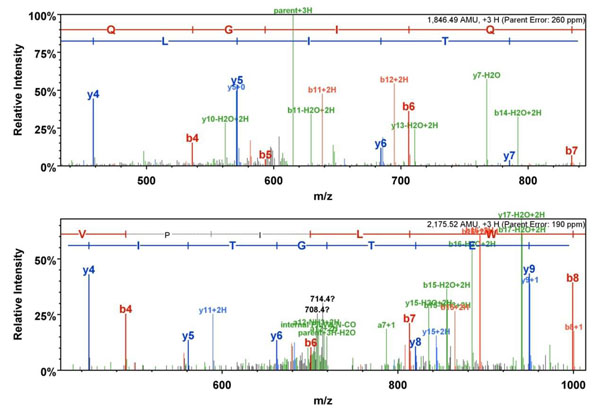
**Multiple-ion spectra analysis.** This figure illustrates the presence of multiple ions types (in green) after *CID*. In the first spectrum, note the presence of *b^o^* and *y^o^* ions with high intensity in the fragmentation of the precursor ion with sequence: FFLQGIQLNTILPDAR, for the protein Lysozyme [Swiss-Prot: P11279]. In the second spectrum, *a*, *b^o^*, b^*^, and *y^o^* ions (all with high intensity) can be observed after the fragmentation of a precursor ion existing in the protein Pratelet glycoprotein 4 [Swiss-Prot P16671].

MS-based methods generally outperform methods using sequence-based learning formulations, as showed by Lee and Singh [3]. However, a number of algorithmic challenges remain outstanding in realizing the potential of MS-based approaches. Salient among these are: (1) *accounting for multiple ion types in the data *[4,5]: To avoid an exponential increase in the search space, a common simplification is to limit the analysis to the spectra of *b*-ions and *y*-ions only [3,6,7]. However, this simplification may erroneously ignore the occurrence of other ions, such as: *a*, b^o^, b^*^, c, *x*, y^o^, y^*^, and *z*. While the occurrence of non-*b/y* ions is minimized (though not eliminated) in collision-induced dissociation (*CID*), some of these ions can be present with greater likelihood in dissociation methods such as electron capture dissociation (*ECD)*, electron transfer dissociation (*ETD),* and electron-detachment dissociation (*EDD)*. In fact these ions types should be considered even in *CID* as illustrated by the example in Figure 2. (2) *Design of efficient search and matching algorithms*: The search space of possible disulfide topologies increases rapidly not only with the number of ion types being analyzed but also with the number of cysteines as well as the types of connectivity patterns. Thus, it is imperative to have algorithms that can accommodate the richness of the entire problem domain. (3) *Automated data-driven determination of parameters:* Many advanced algorithms in this area are intrinsically parametric. Often, determining the optimal value of these parameters automatically is in itself, a complex problem. This places the practitioner at a significant disadvantage. Support for automated and data-driven strategies for estimation of crucial parameters is therefore crucial to the real-world success of a method in this problem domain.

The contributions of this paper in context of the aforementioned challenges include: (1) Development of a highly efficient strategy for multi-ion disulfide bond analysis by considering *a*, *b*, b^o^, b^*^, c, *x*, y, y^o^, y^*^, and *z* ion types. To the best of our knowledge, this is the first algorithmic work that has considered all these ion-types in S-S bond determination. (2) A fully polynomial-time algorithm that selectively generates only those regions of the search space where the correct solutions reside with a high likelihood. (3) A multiple-regression-based data driven method to calculate the critical parameters modulating the search, so as to ensure that the correct bonding topologies are not missed due to the truncation of the search space. At the same time, the parameter selection ensures that the search is focused on the most promising regions of the search-space, and (4) A local-to-global strategy that builds a globally consistent bonding pattern based on MS data at the level of individual bonds.

The proposed approach also implements the probability-based scoring model proposed in [8] for each specific disulfide bond based on the number of MS/MS matches and their respective abundance. These scores reflect the significance of the specific disulfide bond and can form the basis of analysis, such as that conducted in [9], to estimate the accuracy of peptide assignment to tandem mass spectra.

**Figure 3:**
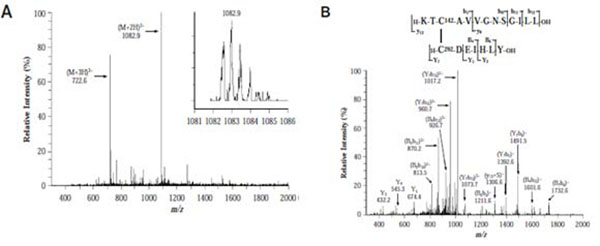
**Two-stage matching spectra for protein ST8SiaIV**. (A) In the first-stage (*DMS* vs. *PMS*), the theoretical disulfide-bonded structure is matched with the doubly charged precursor ion with highest intensity, whose *m/z* = 1082.9. (B) For this initial match, the disulfide-bonded peptide pair is fragmented and the fragments are matched with the MS/MS spectrum for the precursor ion (*FMS* vs. *TMS*), generating a list of validation matches.

At a high-level, the proposed approach can be thought of as a two-stage database-based matching technique (see Figure 3). From this perspective, it shares similarities with [10], where cross-linked peptides were also identified using a two-level method. During the first stage of such two-stage methods, the mass values of the theoretically possible disulfide-bonded peptide structures are compared with precursor ion mass values derived from the MS-spectra. In the second (confirmatory) stage, the theoretical spectra from the disulfide-bonded peptide structures are compared with MS/MS experimental spectra. The confirmatory step is necessary since a disulfide bonded peptide may not actually correspond to a precursor ion, even if their mass values are similar. Our approach can be used to conduct this entire search process in (a low degree) polynomial time. This paper significantly extends our prior research where we had proposed efficient indexing strategies to speed-up the search [11,12] as well as our more recent work [13], where a polynomial time approximation algorithm using hand-crafted parameters was proposed for the first stage matching.

## Methods

We start the description of our method by providing, in Table 1, the key abbreviations used in the ensuing description and their respective definitions. In the first stage of the method, an *Initial Match* (*IM)* is said to be obtained when the difference between the detected mass of a targeted ion from the *PMS* and the calculated mass of a possible disulfide-bonded peptide structure from the *DMS* is found to be less than a threshold *T_IM_*. The second stage validates (or rejects) the initial matches. For each Initial Match, the validation occurs by searching for matches between product ions from the *TMS* and the theoretical spectra *FMS*. A *Validation Match* (*VM*) is said to occur when the difference between a precursor ion fragment mass from *TMS* and a disulfide-bonded fragment structure mass from *FMS* falls below a validation match threshold *T_VM_*.

**Table 1:** Abbreviations and their definitions

Abbreviation	Definition
*DMS*	Set of mass values corresponding to all possible disulfide-bonded peptide structures that can be obtained from a digested protein.
*PMS*	Set of mass values of ions that undergo dissociation to produce product ions (set of precursor ions).
*IM*	Correspondence obtained when the difference between the detected mass of a targeted ion from the *PMS* and the calculated mass of a possible disulfide-bonded peptide structure from the *DMS* is less than a match threshold *T_IM_*.
*T_IM_*	Initial Match threshold. Threshold used to define a mass window centered on a *PMS* value within which a correspondence between a *DMS* value and a *PMS* value may be found.
*ε*	*DMS* trimming parameter used to trim the *DMS* set. To trim the *DMS* set by *ε* means to remove as many elements from *DMS* as possible without losing meaningful mass values.
*TrimSet*	Set of trimmed mass values from the *DMS* set.
*PM*	Peptide Mass: cysteine-containing peptide mass value.
*TempSet*	Temporary mass set containing possible disulfide bonded peptide structures.
*FMS*	Set of mass values of every disulfide-bonded fragment structure that can be obtained from fragment ions, which can be of types *a*, *b*, b^o^, b^*^, c, *x*, y, y^o^, y^*^and *z*.
*TMS*	Set of mass values of the product ions obtained after the MS/MS step (MS/MS spectra).
*VM*	Correspondence obtained when the difference between a precursor ion fragment mass from *TMS* and a disulfide-bonded fragment structure mass from *FMS* falls below a validation match threshold *T_VM_*.
*T_VM_*	Validation Match threshold. Threshold used to define a mass window centered at a *TMS* value in which a correspondence between a *FMS* value and a *TMS* value may be found.
*δ*	*FMS* trimming parameter used to trim the *FMS* set. To trim the *DMS* set by *δ* means to remove as many elements from *FMS* as possible without losing meaningful fragment ions mass values.
*FragSet*	Set containing the mass values of fragment ions generated by the method GENFRAGS(.) in the APROX-FMS routine.

Unfortunately, the sizes of both *FMS* and *DMS* grow exponentially. For a disulfide-bonded peptide structure consisting of *k* peptides, considering that there are *f* different fragment ion types possible, up to *f^k^* types of fragment arrangements may occur in the *FMS*. If the *i*th fragment ion consists of *p_i_* amino acid residues, then the complexity to compute the entire *FMS* for a disulfide-bonded peptide structure is  using a brute-force approach. The *DMS* also grows exponentially. To understand this, let *P = {p_1_, p_2_, …, p_k_}* be the list of cysteine-containing peptides in a polypeptide chain. Further, let *C = {c_1_, c_2_, …, c_i_}* be the list of the number of cysteines per cysteine-containing peptide *p_i_*. If  is the total number of cysteines in a protein, the number of possible disulfide connectivity patterns (*DMS* size) is [1,14]: .

### The subset-sum formulation: towards polynomial-time matching

Given the growth characteristics of the *DMS* and the *FMS*, an exhaustive search-and-match strategy is clearly infeasible in the general case. This is especially true if multiple ion types are considered. Indexing [11,12] and filtering [15] are two possible approaches that have been considered for ameliorating this problem. In this paper we explore an alternative strategy that is based on the key insight that the *entire search space (DMS or FMS) does not need to be generated to determine the matches*. That is, we only want to generate the few disulfide bonded peptides whose mass is close to the (given) experimental spectra rather than generate all possible peptide combinations and subsequently testing and discarding most of these. This insight allows us to re-cast the *DMS* and *FMS* generation as instances of the subset-sum problem [16]. Recall, that given the pair (*S*, *t*), where *S* is a set of positive integers and *t* ∈ ***Z***^+^, the subset-sum problem asks whether there exists a subset of *S* that adds up to *t*. While the subset-sum problem is itself NP-Complete, it can be solved using approximation strategies to obtain near-optimal solutions, in polynomial-time [16].

### Polynomial time DMS mass list construction

Our strategy lies in obtaining an approximate solution to the subset-sum problem by trimming as many elements from *DMS* as possible based on a parameter *ε*. To trim the *DMS* set by *ε* means to remove as many elements from *DMS* as possible such that if DMS^*^ is the resultant trimmed set, then for every element *DMS_i_* removed from *DMS*, there will remain an element DMS_i_^*^ in DMS^*^ which is “sufficiently” close in terms of its mass to the deleted element *DMS_i_*. Specifically,(1)

**Figure 4:**
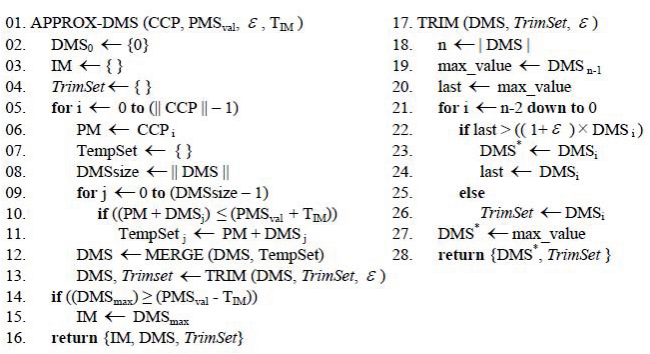
Pseudo code for APROX-DMS and TRIM routines

The approximation algorithm for creating the partial *DMS* is described by the APPROX-DMS and TRIM routines (Figure 4). APPROX-DMS takes the following parameters: (1) a sorted list of cysteine-containing peptides mass values (*CCP*), (2) a target mass value from the *PMS* list (*PMS_val_*), (3) the trimming parameter *ε*, and (4) the Initial Match threshold (*T_IM_*). In lines 2-8 of Figure 4, all the variables and data structures are initialized. In lines 9-11, the theoretical disulfide-bonded peptide structures are formed and stored in a temporary set called *TempSet*. Line 10 excludes values greater than the *PMS_val_* plus a constant corresponding to the Initial Match threshold. The rationale behind this threshold is explained in the following section. Line 12 increments the *DMS* by invoking the routine MERGE, which returns a sorted set formed by merging the two sorted input sets *DMS* and *TempSet*, with duplicated values removed. In line 13, the TRIM routine is called to shorten the *DMS* set. Lines 14-15 examine if the largest mass value in the constructed *DMS* set is sufficiently close to the targeted mass *PMS_val_*. If so, an Initial Match occurs.

Table 2 presents an example showing the effectiveness of the APROX-DMS. In this specific case, 37.5% of the entire search space (all feasible combinations of cysteine-containing peptides) was successfully trimmed, while ensuring that the correct *IM* was not missed. Another example illustrating the action of APPROX-DMS on the Beta-LG protein is available as supplemental information (see Additional File 1).

**Table 2:** Running APROX-DMS on the ST8SiaIV ***C**^142^**-C**^292^*bond

Property	Value
*CCP*	{716, 728, 749, 863, 864, 891, 976, 1096, 1105, 1161, 1204, 1274, 1359, 1367, 1418, 1480, 1593, 1733, 1754, 1846, 1863, 1864, 1976, 2179, 2292, 2351, 2617, 2737, 2822}
*PMS_val_*	{2050.5} *(Precursor ion mass)*
*ε*	0.02530
*T_IM_*	1.0
*DMS*	{728, 749, 863, 891, 976, 1105, 1161, 1204, 1274, 1367, 1418, 1480, 1593, 1639, 1702, 1754, 1846, 1908, 1994, **2050**} – *(value in bold is a valid IM)*
*TrimSet*	{716, 864, 1096, 1359, 1476, 1733, 1863, 1864, 1865, 1867, 1976, 2022}
*IM*	{2050.0} *(KTCAVVGNSGIL – CDEIHLY) – SS-bond: C^142^-C^292^*

*CCP:* the mass values of all cysteine-containing peptides. *PMS_val_*: a disulfide-bonded precursor ion mass. *TrimSet*: all the disulfide-bonded structures trimmed from the set of feasible combinations of cysteine-containing peptides. For this example, 37.5% of the structures were trimmed and the correct *IM* was found.

The complexity of both routines MERGE and TRIM is *O(|DMS|+|TempSet|)* and *O(|DMS|)*, respectively. Further, for any fixed *ε* > 0, our algorithm is a (1 + *ε*)-approximation scheme. That is, for any fixed *ε* > 0, the algorithm runs in polynomial time. The proof of the polynomial time complexity of APPROX-DMS can be obtained by direct analogy to the proof of the polynomial time complexity of the subset sum approximation algorithm from [16] and is outlined in Appendix A.

### Parameters estimation

APPROX-DMS depends on two important parameters, namely, the match threshold *T_IM_* and the trimming parameter *ε*. The match threshold is responsible for defining a “matching window”. This is necessary due to practical considerations such as the sensitivity of the instrument (i.e. 0.01Da, 0.1Da, and 1.0Da) and experimental noise, due to which an exact match is a rarity. We conducted an empirical study by using different values of *T_IM_* for all our datasets. Based on the results, the *T_IM_* value of ±1.0*Da* was found to minimize missing matches as well as the occurrence of false positives. Considering the smallest precursor ion mass involved, in these studies, the above value of *T_IM_* guaranteed a matching accuracy of 99.86%.

The second parameter *ε* is much more important as it is crucial to the running time of the algorithm and its accuracy as evident from Eq. (1). To determine *ε*, we note that it is inversely proportional to the algorithm’s running time. However, a large value of *ε* would cause meaningful fragments to be left out of the *DMS*. At the same time, a small value for *ε* will lead to few data points being trimmed. Thus “guessing” appropriate values of *ε* can be complicated and suboptimal choices can significantly impact the quality of the results. We address the problem of data-driven estimation of *ε* using a regression framework where *ε* is treated as a dependent variable and based on the data, a functional relationship is obtained between it and the other (independent) variables. We model this functional relationship using the following independent variables: (1) the cysteine-containing peptides (*CCP*) mass range defined by *CCP_max_* and *CCP_min_* corresponding to the peptides with the highest and lowest mass respectively. (2) The number of cysteine-containing peptides *k*. A large *k* implies that the average difference in the mass of any two peptide fragments is small. Conversely, a small *k* implies fewer fragments with putatively larger differences in their masses. (3) The cysteine-containing peptides average mass value *CCP_average_*. The relationship between *ε* and these other variables is then obtained using multiple-variable regression. In our studies, the data for the regression was obtained using bootstrapping where groups of four proteins were randomly picked from the set of 9 proteins available to us. The functional relationship defining *ε* was obtained to be:(2)

### Polynomial time FMS construction

In creating the *FMS*, a strategy similar to the one used for generating the *DMS* can be used. This involves using an approximation algorithm, this time, to generate the theoretical spectra for all the *IMs* found during the first-stage matching. We define another trimming parameter *δ* to trim the *FMS* mass list. It can be expected that the functional form of *δ* depends on the fragments mass range, as well as their granularity (extent to which fragments are broken down into smaller ions). In a manner similar to the case for estimating ε, we used regression to obtain the specific functional form for the dependent variable *δ* in terms of the variables *AA_max_* (the largest amino acid residue mass), *AA_min_* (the smallest amino acid residue mass), *AA_average_* (the average amino acid residues mass), and *||p||* (average number of amino acid residues per fragment). Bootstrapping was once again utilized, resulting in the relationship shown in Eq. (3).(3)

**Figure 5:**
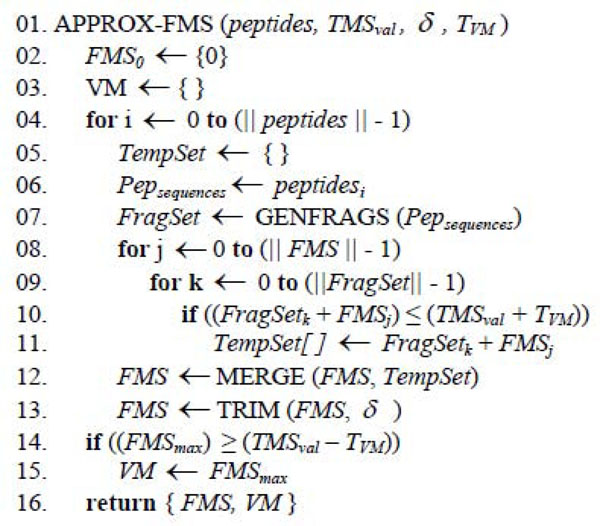
Pseudo code for APROX-FMS routine

The pseudocode of the APPROX-FMS procedure used for generating the *FMS* is shown in Figure 5. The function GENFRAGS(.), in line 7, generates multiple fragment ions (*a*, *b*, b^o^, b^*^, c, *x*, y, y^o^, y^*^, and *z*) for peptide sequences in *Pep_sequences_*, which contains the disulfide-bonded peptides involved in the *IM* being analyzed. Next, for each element in the *FMS* and for each fragment in the *FragSet* (lines 8-11), new disulfide-bonded peptide fragment structures are formed. Line 10 rejects values greater than the *TMS_val_*, considering the Validation Match threshold. In line 12, the current *FMS* set is combined with the disulfide-bonded peptide fragments set *TempSet* using MERGE. In line 13, the *FMS* is trimmed using the TRIM routine. Lastly, a Validation Match *VM* is declared (lines 14-15) when a correspondence is found between the mass of the largest value in *FMS* and an experimentally determined mass value *TMS_val_*, given a Validation Match threshold.

### Determining the globally consistent bond topology

Once all the *Initial Matches* and *Validation Matches* are calculated, we have a “local” (putative bond-level) view of the possible disulfide connectivity. This local information needs to be integrated to obtain a globally consistent view. Our approach to this problem is motivated by Fariselli and Casadio [14]. Specifically, we model the location of the putative disulfide bonds by edges in an undirected graph *G* (*V*, *E*), where the set of vertices *V* corresponds to the set of cysteines. To each edge, we assign a match score. This score represents the combined importance of each single peak match within two spectra. Each specific peak match is weighted according to its intensity. The match score is given by:(4)

In Eq. (4), the numerator corresponds to the sum of each validation match for a disulfide bond multiplied by the matched MS/MS fragment normalized intensity value (*I_N_*). Here, *VM_i_* is a binary value which is set to 1 if a confirmatory match was found for fragment *i*. The denominator similarly contains the sum of each experimental MS/MS fragment ion from *TMS* multiplied by *I_N_*. Here*, TMS_i_* is a binary variable which indicates the presence of a fragment *i* in the MS/MS spectrum.

Next, the globally consistent bond topology is found by solving the maximum weight matching problem for the graph *G*. A matching *M* in the graph *G* is a set of pair-wise non-adjacent edges; that is, two edges do not share a common vertex. A maximum weight matching is defined as a matching *M* that contains the largest possible sum of the weights (match scores) of each possible edge (disulfide bond). We use the Gabow algorithm [17], as implemented in [18] for computing the maximum weight match.

## Results

The proposed method was validated utilizing experimental data obtained using a capillary liquid chromatography system coupled with a Thermo-Fisher LCQ ion trap mass spectrometer LC/ESI-MS/MS system. Details of the experimental protocols can be found in [19,20]. We used data from nine eukaryotic glycosyltransferases. These molecules and their Swiss-Prot ID were: ST8Sia IV [Q92187], Beta-lactoglobulin [P02754], FucT VII [Q11130], C2GnT-I [Q09324], Lysozyme [P00698], FT III [P21217], β1-4GalT [P08037], Aldolase [P00883], and Aspa [Q9R1T5].

We conducted five sets of experiments to investigate the proposed method and its efficacy. These experiments included: (1) Analysis of method’s efficiency, showing how the method successfully reduced the *DMS* and *FMS* search spaces. (2) Analysis of the effect of incorporating multiple ion types, demonstrating the importance of considering non-*b*/*y* ions in the determination of disulfide bonds. (3) Comparative analysis of the proposed method with established predictive techniques. (4) Comparative analysis of the method with MassMatrix, an established MS-based approach which can be used for determining S-S bonds. In both experiment 3 and experiment 4, the aforementioned set of glycosyltransferases and their known S-S bond topology provided us with the ground truth. (5) Analysis of the method in terms of established performance measures: *Accuracy* (*Q_2_*), *Sensitivity* (*Q_c_*), *Specificity* (*Q_nc_*), and *Matthew’s correlation coefficient (c)*.

### Analysis of efficiency of the search

One of the most important characteristics of the proposed method is its efficiency in terms of excluding significant portions of a large and rapidly expanding search space. In Table 3 we compare the size of the complete *DMS* (containing all the disulfide-bonded peptide structures generated for each protein) and the complete *FMS* (containing all the disulfide-bonded fragment ions) with the truncated *DMS* and *FMS* obtained using the proposed approach.

**Table 3:** DMS and FMS mass space sizes comparison

Protein	Disulfide Bond	Full Search (exponential)		Proposed Search (polynomial)		DMS decrease	FMS decrease
		DMS size	FMS size	DMS size	FMS size		
Beta-LG	C^82^C^176^	2152	2169	1870	78	13.1%	96.4%

ST8Sia IV	C^142^C^292^	1246	1792	1038	106	16.7%	94.1%
	C^156^C^356^	1246	2640	1038	255	16.7%	90.3%

FucT VII	C^318^C^321^	581	115	528	34	9.1%	70.4%
	C^68^C^76^	879	103	681	41	22.5%	60.2%
	C^211^C^214^	879	1819	681	107	22.5%	94.1%

B1,4-GalT	C^134^C^176^	2149	1189	1127	77	47.6%	93.5%
	C^247^C^266^	2149	5480	1127	426	47.6%	92.2%

Average DMS and FMS decrease						21.8%	86.4%

**Figure 6:**
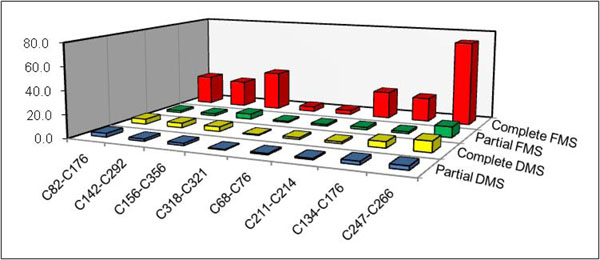
**Comparison of the computational time (in seconds) for the exhaustive and partial generation of *DMS* and *FMS* of the proteins from** Table 3. On average there was a 49.5% decrease in time to compute the *DMS* and 88.7% decrease in time to compute the *FMS*. The computations were carried out on an Intel T2390 1.86 GHz single-core processor with 1GB RAM.

It may be noted that across the molecules, on an average, the proposed approach required examining about 78% of the entire *DMS* and only about 14% of the entire FMS. It is crucial to note that this reduction in search was achieved without impacting the accuracy *and* having considered all multiple fragment ion types (*a*, *b*, b^o^, b^*^, c, *x*, y, y^o^, y^*^, and *z*). The *DMS* decrease was less than the *FMS decrease* because the disulfide-bonded structures in the *DMS* were bigger and fewer in number and consequently dispersed across the spectra mass range. In Figure 6, we show the actual time taken to obtain a solution by generating the complete *DMS* and *FMS*, as well as their truncated counterparts, for each of the molecules.

### Effects of incorporating multiple ion types: a case study

In this experiment, we investigated the effect of incorporating multiple ion types (*a*, *b*, b^o^, b^*^, c, *x*, y, y^o^, y^*^, and *z*) in determining the S-S bonds as opposed to considering only *b*/*y*-ions. We found that multiple instances of combinations between *b*/*y* ions and other ions types occurred by analyzing the confirmatory matches for the different disulfide bonds. These combinations are available as supplemental information (see Additional File 2).

**Figure 7:**
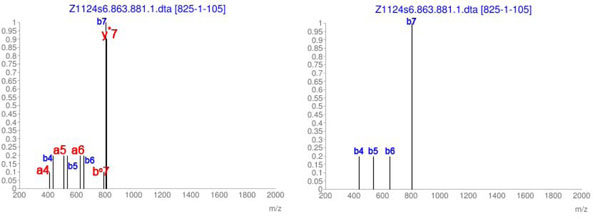
**Spectra samples from tryptic digested protein FucT VII**. Spectra (m/z vs. normalized intensity) illustrating the confirmatory matches (whose intensity values were at least 10% of the maximum intensity) found for the disulfide bond between cysteines C^318^-C^321^ in protein FucT VII. The spectrum in the left shows the matches found when multiple ions were considered. The spectrum in the right shows the matches when only *b*/*y*-ions were considered.

The consideration of multiple ion types also contributed to the method’s accuracy in terms of determining specific S-S bonds. Disulfide bonds previously missed due to their low match score could be identified when all ten different ion types were considered. The tryptic-digested protein FucT VII (which underwent *CID*) constituted one such example. In FucT VII the bond C^318^-C^321^ was missed when considering only *b*/*y* ions (match score 29, *pp*=11, *pp2* =15). However, as shown in Figure 7, this bond was identified when multiple ions types were included (match score 100, *pp*=31, *pp*2=70). The confidence measures *pp* and *pp2* are described in the following section. To explain this improvement we note that C^318^-C^321^ was an intra-bond involving cysteines that were close together. Consequently, CID-based fragmentation was poor and the consideration of other ion types essentially improved the signal-to-background contrast. In this particular case, five other ion types - *a_4_*, *a_5_*, *a_6_*, *b^o^_7_*, y^*^_7_ - were present in the FucT VII MS/MS data besides the *b* ions represented in the spectrum on the right in Figure 7. In the following, we present details of how these ions contribute to the match score *V_s_* (from Eq. (4)). We present the two cases: consideration of only *b/y*-ions (Eq. (5)) and consideration of multiple ion types (Eq. (6)). In the numerator we specify the contribution of each spectrum peak from Figure 7 (the ion corresponding to each *VM_i_ × I_N_* term is showed in brackets).(5)(6)

We also observed that consideration of multiple ion-types led to significant increase in the match scores of the true disulfide bonds, whereas only a modest increase was noticed for false positives. This allowed us to increase the threshold we use on the match score *V_s_* to identify high-quality matches from 30 to 80 (a 166% increase). The positive effect of this increment on the specificity of the method can be illustrated by considering the protein Aldolase. In this molecule, consideration of only *b*/*y* ions led to a false positive S-S bond identification between cysteines C^135^-C^202^ (*V_s_*=30.8, with (original) threshold 30) However, when the multiple ions-types were considered with the (increased) threshold on the match score, no S-S bond was found between C^135^-C^202^ (*V_s_*= 53.2, (incremented) threshold 80).

### Comparative studies with predictive techniques

In this experiment we compared the proposed method with three well known predictive methods DiANNA [21], DISULFIND [22], and PreCys [23]. The results from each of the methods are shown in Table 4 along with the with the known disulfide bond linkages according to the Swiss-Prot knowledgebase. As it can be seen, in terms of correct identifications (as well as minimizing false positives), the proposed approach outperformed all the predictive techniques.

**Table 4:** Comparison with predictive methods

Protein	Known Pattern	Proposed Algorithm	DiANNA 1.1	DISULFIND	PreCys
ST8Sia IV	C^142^C^292^, C^156^C^356^,	**C^142^C^292^, C^156^C^356^**	C^11^C^156^, C^142^C^292^, C^169^C^356^	None	C^142^C^356^, C^156^C^292^
Beta-LG	C^82^C^176^, C^122^C^135^	**C^82^C^176^**	C^12^C^137^, C^82^C^176^, C^126^C^135^	None	None
FucT VII	C^68^C^76^, C^211^C^214^, C^318^C^321^	**C^68^C^76^, C^211^C^214^, C^318^C^321^**	C^68^C^321^, C^76^C^211^, C^214^C^318^	C^76^C^318^	C^68^C^76^, C^211^C^214^, C^318^C^321^
B1,4-GalT	C^134^C^176^, C^247^C^266^	**C^134^C^176^, C^247^C^266^**	C^23^C^176^, C^30^C^144^, C^266^C^341^	None	C^134^C^247^, C^176^C^266^
C2GnT-I	C^59^C^413^, C^100^C^172^, C^151^C^199^, C^372^C^381^	**C^59^C^413^, C^151^C^199^, C^372^C^381^**	C^13^C^172^, C^59^C^217^, C^151^C^234^, C^199^C^372^, C^381^C^413^	Not supported	C^59^C^381^, C^100^C^372^, C^151^C^172^, C^199^C^413^
Lysozyme	C^24^C^145^, C^48^C^133^	**C^24^C^145^, C^48^C^133^,**	C^24^C^145^, C^48^C^133^, C^82^C^98^, C^94^C^112^	C^24^C^145^, C^48^C^133^, C^82^C^98^, C^94^C^112^	C^82^C^145^
FT III	C^81^C^338^, C^91^C^341^	**C^81^C^338^**	C^16^C^91^, C^81^C^143^, C^129^C^338^	None	C^81^C^91^
Aldolase	None	**None**	C^73^C^339^, C^135^C^290^, C^115^C^240^, C^178^C^202^	None	None
Aspa	None	**None**	C^4^C^275^, C^60^C^217^, C^66^C^151^, C^123^C^145^	None	None

### Comparative studies with MassMatrix

At the state-of-the-art MS2Assign [6] and MassMatrix [7] are two MS-based methods that can be applied to the problem of determining S-S bond connectivity. In our previous work [3], the MS2DB system developed by us was found to be comparable to MS2Assign [6], albeit, in limited testing. Since the proposed method improves upon MS2DB and due to space limitations, we only present detailed comparative results with MassMatrix [7] in Table 5. As part of this experiment, for each S-S bond, in addition to the empirical match score (Eq. (4)), a probability based scoring model proposed in [8] was implemented. This model provided two scores called *pp* and *pp2* scores. The *pp* score helps to evaluate whether the number of *VMs* could be a random. The *pp2* score evaluates whether the total abundance (intensity) of *VMs* could be a random. We refer the reader to [8] for a detailed description and formulae of the *pp* and *pp2* scores. The reader may note that the proposed method had better *pp* and *pp2* scores when compared to MassMatrix (higher *pp* and *pp2* scores are better, indicating smaller *p*-values). While the match scores (*V_s_*) obtained with the proposed method were also higher than those obtained with MassMatrix (V^*^_s_), no inferences should be drawn as these scores are calculated differently in each of these methods. As can be seen from Table 5, every bond correctly determined by MassMatrix was also found by us. However, there were S-S bonds in C2GnT-I and Lysozyme that were found by the proposed method but not by MassMatrix.

**Table 5:** Comparison with MassMatrix

Protein	Known Pattern	Proposed Method	MassMatrix
ST8Sia IV	C^142^C^292^, C^156^C^356^	**C^142^C^292^** [*V_s_:131;pp:109;pp2:41*]**, C^156^C^356^** [*V_s_:100;pp:97;pp2:6*]	C^142^C^292^ [V^*^_s_:54;pp:15;pp2:13]**,** C^156^C^356^ [V^*^_s_:77;pp:23;pp2:15]
Beta-LG	C^82^C^176^, C^122^C^135^	**C^82^C^176^** [*V_s_:100;pp:49;pp2:16*]	C^82^C^176^ [V^*^_s_:68;pp:14;pp2:14]
FucT VII	C^68^C^76^, C^211^C^214^, C^318^C^321^	**C^68^C^76^** [*V_s_:105;pp:41;pp2:98*]**, C^211^C^214^** [*V_s_:100;pp:13;pp2:20*]**, C^318^C^321^** [*V_s_:100;pp:31;pp2:70*]	C^68^C^76^ [V^*^_s_:12;pp:9;pp2:3], C^211^C^214^ [V^*^_s_:78;pp:16;pp2:11], C^318^C^321^ [V^*^_s_:46;pp:28;pp2:16]
B1,4-GalT	C^134^C^176^, C^247^C^266^	**C^134^C^176^** [*V_s_:100;pp:61;pp2:29*]**, C^247^C^266^** [*V_s_:195;pp:88;pp2:177*]	C^134^C^176^ [V^*^_s_:34;pp:9;pp2:7], C^247^C^266^ [V^*^_s_:31;pp:7;pp2:7]
C2GnT-I	C^59^C^413^, C^100^C^172^, C^151^C^199^, C^372^C^381^	**C^59^C^413^** [*V_s_:158;pp:237;pp2:61*]**, C^151^C^199^** [ *V_s_:100;pp:93;pp2:15*]**, C^372^C^381^** [*V_s_:100;pp:81;pp2:79*]	None
Lysozyme	C^24^C^145^, C^48^C^133^	**C^24^C^145^** [*V_s_:140;pp:65;pp2:88*]**, C^48^C^133^** [*V_s_:100;pp:62;pp2:55*]	C^48^C^133^ [V^*^_s_:135;pp:51;pp2:33]
FT III	C^81^C^338^, C^91^C^341^	**C^81^C^338^** [*V_s_:100;pp:179;pp2:93*]	None
Aldolase	None	**None**	None
Aspa	None	**None**	None

The score (V_s_) of each disulfide bond and the confidence scores (*pp* and *pp2* values*)* are shown in brackets, respectively.

### Quantitative assessment and analysis of the method’s performance

If the set of disulfide bonds are denoted by *P* and the set of cysteines not forming disulfide bonds by *N*, then true positive (*TP*) predictions occur when disulfide bonds that exist are correctly predicted. False negative (*FN*) predictions occur when bonds that exist are not predicted as such. Similarly, a true negative (*TN*) prediction correctly identifies cysteine pairs that do not form a bond. Finally, a false positive (*FP*) prediction, incorrectly assigns a disulfide link to a pair of cysteines, which are not actually bonded. Based on these definitions, we use the following four standard measures to analyze the proposed method.

Sensitivity (Q_c_) = *TP/P* (7)

Specificity (Q_nc_) = *TN/N* (8)

Accuracy (Q_2_) = *TP + TN/P + N* (9)

In Table 6 we present the results obtained for our framework. With maximum specificity and high accuracy (98% average), the method correctly reported the connectivity for most of the proteins. The method only failed to identify three disulfide bonds. One intra-bond in the Beta-LG protein could not be found due to a blind spot caused by the same intra-bond, making the protein’s fragmentation difficult. A *blind spot* occurs when the precursor ion fragmentation produces different fragments only at the outside boundaries of the intra-disulfide bond. This can cause too few product ions to be generated; the limited information can prevent accurate determination of disulfide bonds using MS-based methods. One cross-linked bond in the FT III protein also could not be identified because this particular connectivity configuration creates a large disulfide-bonded structure, which is poorly fragmented by tandem mass spectrometry. One bond in the C2GnT-I protein could not be found, since the precursor ion cannot be formed by chymotryptic digestion, which was the digestion carried for C2GnT-I. It is important to note that neither MassMatrix nor MS2Assign were able to identify these bonds.

**Table 6:** Sensitivity, specificity, accuracy and Mathew’s correlation coefficient results for all nine proteins analyzed

Protein	*Q_c_*	*Q_nc_*	*Q_2_*	*c*
ST8Sia IV	1.00	1.00	1.00	1.00
Beta-LG	0.50	1.00	0.95	0.69
FucT VII	1.00	1.00	1.00	1.00
C2GnT-I	0.75	1.00	0.98	0.86
Lysozyme	1.00	1.00	1.00	1.00
B1,4-GalT	1.00	1.00	1.00	1.00
FT III	0.50	1.00	0.94	0.69
Aldolase	X	1.00	1.00	X
Aspa	X	1.00	1.00	X

## Conclusions

We have presented an algorithmic framework for determining S-S bond topologies of molecules using MS/MS data. The proposed approach is computationally efficient, data driven, and has high accuracy, sensitivity, and specificity. It is not limited either by the connectivity pattern or by the variability of product ion types generated during the fragmentation of precursor ions. Furthermore, the approach does not require user intervention and can form the basis for high-throughput S-S bond determination.

## Authors' contributions

The algorithmic solution framework was designed by RS and implemented by WM. Computational studies and experiments were carried out by WM and RS. T-YY developed the experimental protocols and generated the data. The paper was written by RS and WM.

## Competing interests

The authors declare that they have no competing interests.

## Appendix A – Etudes of the proof of polynomial complexity

The proof that the proposed method is a fully polynomial approximation scheme consists of two parts. First, we need to show that each value returned by the APPROX-DMS function is within 1 + *ε* from the optimal solution. Second, we need to show that the running time of the method is fully polynomial. We refer the reader to [16] for the proof of the first part and focus in the following on analyzing the complexity of the method. To show that the method is a fully polynomial-time approximation scheme, we derive a bound on the length of a *DMS* set. After trimming, successive elements *DMS_i_* and  of *DMS* must have a relationship . Therefore, each possible *DMS* set contains up to log_1+ε_*PML_val_* values. Since (*x*/(1 + *x*)) ≤ ln(1 + *x*) ≤ *x* and 0 < ε < 1, it can be shown that:(11)

As can be seen from Eq. (11), this bound is (explicitly) polynomial in the size of the input *PMS_val_*. It is also (implicitly) polynomial in the size of the set *DMS* since ε is directly proportional to the number of cysteine-containing peptides *k* (per Eq. (2)) and these peptides are in turn combined to form each element of the *DMS*. A similar argument can be made for the APPROX-FMS routine, completing thereby the proof that the proposed method is a fully polynomial-time approximation scheme.

## Supplementary Material

Additional File 1 **Action of APPROX-DMS on the protein Beta-LG** This example shows the effectiveness of the APROX-DMS algorithm while trimming a *DMS* set generated for the protein Beta-LG using MS/MS data. Click here for file

Additional File 2 **Combination between *b*/*y* ions and other ions types on MS/MS data** This example shows that combinations between ion types other than just *b* and/or *y* ions do occur, even for proteins that underwent *CID* (*CID* is a dissociation method which produces mainly *b/y* ions). Click here for file
