# Supplementary material for: An efficient algorithmic approach for mass spectrometry-based disulfide connectivity determination using multi-ion analysis
Source: BMC Bioinformatics. 2011 Feb 15;12(Suppl 1):S12. doi: 10.1186/1471-2105-12-S1-S12 (PMC3044266; doi:10.1186/1471-2105-12-S1-S12)
Supplement: Additional File 1 — Action of APPROX-DMS on the protein Beta-LG This example shows the effectiveness of the APROX-DMS algorithm while trimming a DMS set generated for the protein Beta-LG using MS/MS data. [file 1471-2105-12-S1-S12-S1.pdf]

<u>Action of APPROX-DMS on the protein Beta-LG</u>

An example illustrating the action of APPROX-DMS on the Beta-LG protein is shown below. The table presents snapshots of the *DMS*, *TrimSet*, and *IM* sets for each CCP_i (cysteine-containing peptide) mass value during each iteration of the trimming process.

Supplementary table 1. *DMS*, *TrimSet* and *IM* mass sets for each CCP_i mass value generated from the tryptic digestion of the protein Beta-lactoglobulin (Beta-LG). The values presented here are retrieved at the end of each loop cycle (lines 5-13) in Figure 4.

i	CCP_i	DMS	TrimSet	IM
0	1064	{0,1064}	{}	{}
1	1192	{0,1064, 1192, 2254}	{}	{}
2	1658	{0, 1064, 1192, 1658, 2254, 2720, 2849}	{}	{}
3	1746	{0, 1064, 1192, 1658, 1746, 2254, 2720, 2849, 2937}	{2809}	{}
4	2275	{0, 1064, 1192, 1658, 1746, 2275, 2720, 2849, 2937}	{2254, 2809}	{}
5	2477	{0, 1064, 1192, 1658, 1746, 2275, 2477, 2720, 2849, 2937}	{2254, 2809}	{}
6	2535	{0, 1064, 1192, 1658, 1746, 2275, 2535, 2720, 2849, 2937}	{2254, 2477, 2809}	{}
7	2648	{0, 1064, 1192, 1658, 1746, 2275, 2535, 2648, 2720, 2849, 2937}	{2254, 2477, 2809}	{}
8	2776	{0, 1064, 1192, 1658, 1746, 2275, 2535, 2648, 2776, 2849, 2937}	{2254, 2477, 2720, 2809}	{}
9	2790	{0, 1064, 1192, 1658, 1746, 2275, 2535, 2648, 2776, 2849, 2937}	{2254, 2477, 2720, 2790, 2809}	{}
10	2930	{0, 1064, 1192, 1658, 1746, 2275, 2535, 2648, 2776, 2849, 2937}	{2254, 2477, 2720, 2790, 2809, 2930}	{}
11	3189	{0, 1064, 1192, 1658, 1746, 2275, 2535, 2648, 2776, 2849, 2937, 3189}	{2254, 2477, 2720, 2790, 2809, 2930}	{3189}
