# Supplementary material for: An efficient algorithmic approach for mass spectrometry-based disulfide connectivity determination using multi-ion analysis
Source: BMC Bioinformatics. 2011 Feb 15;12(Suppl 1):S12. doi: 10.1186/1471-2105-12-S1-S12 (PMC3044266; doi:10.1186/1471-2105-12-S1-S12)
Supplement: Additional File 2 — Combination between b/y ions and other ions types on MS/MS data This example shows that combinations between ion types other than just b and/or y ions do occur, even for proteins that underwent CID (CID is a dissociation method which produces mainly b/y ions). [file 1471-2105-12-S1-S12-S2.pdf]

<u>Combination between *b*/*y* ions and other ions types on MS/MS data</u>

In the following, we show that combinations between ion types other than just *b* and/or *y* ions do occur, even for proteins that underwent *CID*. Most importantly, many of these combinations were present with high abundance across the proteins we analyzed.

Supplementary table 2. Different combinations of multiple ion types present in some of the proteins we used to validate the method proposed. For each protein, we present all combinations involving ion types other than just *b* and/or *y* ions, whose normalized intensity (or abundance) was found to be at least 50% of the highest intensity value measured.

Protein	Disulfide Bonds	Confirmatory matches (each match is presented as a [ion types; abundance measured] pair)
ST8Sia IV	$C^{142}C^{292}$	[$b^*_3b^o_5$; 56%], [$b^*_3a_7$; 100%], [$c_4b^o_5$; 80%], [$b_5y^o_{12}$; 62%]
	$C^{156}C^{356}$	[$z_7b^*_5$; 66%], [$a_8b^*_4$; 75%], [a_8a_4; 69%], [y_4a_{10}; 63%]
Beta-LG	$C^{82}C^{176}$	[a_{10}; 68%], [c_{11}; 51%], [$x_5y^*_6$; 58%], [x_5y_{14}; 100%], [b_6c_{13}; 68%], [$c_8y^*_{14}$; 66%]
FucT VII	$C^{68}C^{76}$	[y^*_7; 57%], [a_5a_{15}; 100%], [$b^*_7b^*_{14}$; 57%]
	$C^{211}C^{214}$	[b^o_9; 75%]
	$C^{318}C^{321}$	[z_7; 100%], [y^*_7; 95%]
B1,4-GalT	$C^{134}C^{176}$	[a_{18}; 63%], [$b^*_7a_{18}$; 76%]
	$C^{247}C^{266}$	[c_5c_{24}; 100%]
C2GnT-I	$C^{59}C^{413}$	[b^*_{14}; 100%], [$x_7y^*_7$; 84%], [$z_3y^o_{11}$; 56%], [$z_3y^*_{11}$; 79%], [a_{16}; 77%], [z_6c_{16}; 77%], [y_9c_{18}; 93%], [$x_{16}y_{13}$; 79%]
	$C^{372}C^{381}$	[c_6x_8; 100%], [x_{23}; 52%], [c_9a_{20}; 100%], [$c_6b^o_{22}$; 98%], [$c_6b^*_{22}$; 50%]
	$C^{151}C^{199}$	[a_9; 100%], [a_3c_9; 51%], [$b^*_7b^o_{10}$; 72%], [$b^*_7y_9$; 100%], [$a_3b^*_{14}$; 65%], [$b^*_4a_{14}$; 78%]
Lysozyme	$C^{24}C^{145}$	[x_3a_6; 100%]
	$C^{48}C^{133}$	[$x_2y^o_4$; 100%], [a_{11}; 50%]
